# Antiprolactinoma Effect of Hordenine by Inhibiting MAPK Signaling Pathway Activation in Rats

**DOI:** 10.1155/2020/3107290

**Published:** 2020-04-23

**Authors:** Xiong Wang, Run-zhu Guo, Li Ma, Qiao-yan Ding, Jun-hua Meng, Yong-gang Chen, Jin-hu Wu

**Affiliations:** ^1^Department of Pharmacy, Tongren Hospital Affiliated to Wuhan University (The Third Hospital of Wuhan), Wuhan, Hubei, China; ^2^Department of Pharmacy, Wuhan Asia Heart Hospital, Wuhan, Hubei, China; ^3^College of Pharmacy, Hubei University of Chinese Medicine, Wuhan, Hubei, China

## Abstract

Prolactinomas are harmful to human health, and the clinical first-line treatment drug is bromocriptine. However, 20% prolactinomas patients did not respond to bromocriptine. Hordenine is an alkaloid separated from Fructus Hordei Germinatus, which showed significant antihyperprolactinemia activity in rats. The aim of this study was to explore the effect and mechanism of hordenine on prolactinomas in rats. The study used estradiol to induce prolactinomas, which caused the activation of the pituitary mitogen-activated protein kinase (MAPK) pathway in rats significantly. The treatment of hordenine restored estradiol, induced the overgrowth of pituitary gland, and reduced the prolactin (PRL) accumulation in the serum and pituitary gland of rats by blocking the MAPK (p38, ERK1/2, and JNK) activation and production of inflammatory cytokines, tumor necrosis factor-*α* (TNF-*α*), interleukin-1*β* (IL-1*β*), and interleukin-6 (IL-6). The antiprolactinoma effect of hordenine was mediated by inhibiting the MAPK signaling pathway activation in rats.

## 1. Introduction

As a benign adenomas, pituitary tumor accounts for 15% of all intracranial neoplasms [1, 2]. The prevalence of pituitary tumor is relatively high in the general population, with approximately 77 cases per 100.000 [3, 4] and reached to a 20% prevalence in clinical occult pituitary adenomas [5]. In the clinic, prolactinomas are the most frequent pituitary tumors, which account for 50% [6]. Prolactinomas are harmful to human body and usually causes severe symptoms such as hyperprolactinemia. The excessive PRL secretion by hyperprolactinemia often results in galactorrhea, decreased libido, and even infertility, especially in women. Although prolactinomas are benign, macroprolactinomas seem to be more resistant to drug therapy and aggressive than microprolactinomas [7]. At present, the pathogenesis theory of prolactinomas includes hypothalamic dysregulation and cancer gene mutation, but the exact pathogenesis is still unknown.

At present, the principle of treating prolactinomas is to decrease excessive serum PRL levels and to reduce tumor size, at last to restore pituitary function in patients. Under physiological conditions, prolactin production of the pituitary gland is inhibited by dopamine, which mainly acts on the dopamine D2 receptors (Drd2) expressed on the membranes of lactotroph cells [8]. In the clinic, most of prolactinomas patients show an effective response to the dopamine agonists (DAs) therapy such as bromocriptine and cabergoline, which are the first-line therapies compared with the surgical therapy [9]. However, 25% of prolactinoma patients do not respond to DA therapy, even at high doses of DA [10]. Furthermore, cabergoline is not on sale in China and other countries at present. Therefore, these problems bring a great challenge to the clinical treatments.

As a classical signal molecule, the mitogen-activated protein kinase (MAPK) pathway plays important roles from extracellular signals to intracellular responses. In various diseases including cancers, the changes in MAPK signal transduction were found. More, the complex signaling cascades of MAPK were involved in tumorigenesis, development, and drug resistance [11]. The MAPK family includes many kinds of kinases, which often undergo changes and results in cancers. MAPKs include p38 MAPK, extracellular signal-regulated kinase (ERK1/2), and c-jun-N-terminal kinase (JNK1/2) [12]. In pathological conditions, extracellular stresses induce the production of cytokines and chemokines, resulting in the MAPK activation [13]. MAPK activation promoted the formation of inflammation and associated cancer [14]. Many studies have reported that p38 MAPK participated in the development and progress of lung cancer [15], prostate cancer [16–18], bladder cancer [19, 20], breast cancer [21, 22], liver cancer [23], transformed follicular lymphoma [24, 25], and leukemia [26]. Furthermore, p38*γ* showed a protumorigenic role for skin carcinogenesis [27]. And, p38*γ* regulated the oncogenic protein K-Ras of colorectal cancer [28]. Studies indicated that MAPK kinases can also inhibit tumor formation and progress [29]. MAPK are implicated in linking the inflammation and tumor development.

**Figure 1:**
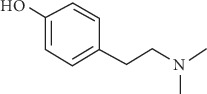
The structure of hordenine

Hordenine is an alkaloid separated from traditional Chinese herb Fructus Hordei Germinatus. The structure of hordenine is shown in Figure 1. In previous study, we have reported that water extract and total alkaloids of Fructus Hordei Germinatus showed antihyperprolactinemia activity significantly [30, 31]. Furthermore, hordenine showed significant antihyperprolactinemia activity in rats [32]. This study was the first to study the possible mechanisms by which hordenine improved these abnormalities.

## 2. Materials and Methods

### 2.1. Materials

Bromocriptine was bought from Gedeon Richter Ltd. (Budapest, Hungary). Hordenine was bought from Chengdu Must Bio-Technology Co. Ltd. (Chengdu, PR China). The antibodies of prolactin (PRL), tumor necrosis factor-*α* (TNF-*α*), interleukin-1*β* (IL-1*β*), and interleukin-6 (IL-6) were obtained from Affinity Biosciences (Cincinnati, USA). The antibodies of nuclear factor kappa-B (NF-*κ*B), p38, p-p38, JNK, p-JNK, ERK1/2, and p-ERK1/2 for rats were supported by Proteintech Group, Inc (Wuhan, PR China). The PRL enzyme-linked immunosorbent assay (ELISA) kit was bought from Wuhan Huamei Bioengineering Co., Ltd. (Wuhan, China).

### 2.2. Establishment of Prolactinoma Model Animals and Experimental Protocol

Eighty female F344 rats (weighing 250–300 g) were purchased from Beijing Wei Tong Li Hua Laboratory Animal Technology Co., Ltd. (Beijing, PR China). Rats were housed in a standard animal room at 22–25°C with a relative humidity of 60 ± 5%. And, they adapted to the environment of laboratory for one week before starting experiments.

Rats were randomly separated into seven groups: control group, sham-operated group (ovariectomized), model group (sham + estradiol injection), bromocriptine group (sham + estradiol injection + 0.393 mg/kg of bromocriptine, as positive control group), and hordenine (sham + estradiol injection + 38.2, 76.4 or 152.8 mg/kg of hordenine) groups (*n* = 10). Except control rats, other F344 rats were all ovariectomized. Except control group and sham-operated group rats, the abdominal cavity of rats was injected with 17*α*-estradiol according to the dosage of 1 mL (2 mg) for every rat. It was carried out once every five days and lasted for 50 days. After 50-day injection of estradiol, hordenine was all dissolved in water and given to rats by means of intragastric administration daily at 9 : 00–10 : 00 a.m. for 30 days, respectively. The administration of drug dose was calculated according to the previous preclinical experiment data. Food and water intake was recorded daily.

### 2.3. Nuclear Magnetic Resonance (NMR) Imaging Detection

Appearance imaging of pituitary gland of rats was detected and recorded by animal NMR. And, the volume of pituitary gland in rats was calculated quantitatively by analytic software (Carimas 2.9).

### 2.4. Serum PRL Level Test

Blood was collected from the femoral artery of rats, and then the serum was separated immediately. For PRL ELISA, 2 *μ*l of serum was coated in a 96-well plate overnight at 4°C. After being washed with 0.1% of Tween, samples were added with 10% of BSA solution, followed by avidin and biotin. The biotinylated hyaluronan-binding protein (Wuhan, Huamei) was then added into the samples and incubated for one hour at the room temperature. After the samples were washed for three times, avidin-horseradish peroxidase was added for incubation. 2,2-Azinobis (3-ethylbenzthiazoline-6-sulfonic acid) was used and set as a substrate, and the absorbance was set and read at 405 nm.

### 2.5. Western Blot Analysis

After oral administration of drugs, rats were executed by carbon dioxide and the pituitary glands were excised, which were then immediately flash-frozen and stored at −80°C. Frozen tissue was weighed and then placed in lysis buffer (pH 7.5 of Tris, 150 mM of NaCl, and 1% of NP-40) with protease inhibitors to prepare the tissue protein lysates. Tissue homogenization was prepared with a homogenizer (Wuhan Servicebio). Sample proteins were separated on either 10 or 12% of SDS-PAGE gels. Gels were transferred to polyvinylidene fluoride membranes, and 5% milk was added to block. Then, the membranes were incubated at 4°C overnight with primary antibodies: rabbit anti-rat PRL (Affinity Biosciences), rabbit anti-rat TNF-*α* (Affinity Biosciences), rabbit anti-rat IL-6 (Affinity Biosciences), rabbit anti-rat IL-1*β* (Affinity Biosciences), rabbit anti-rat NF-*κ*B (Proteintech Group Inc.), rabbit anti-mouse p38 (Proteintech Group Inc.), rabbit anti-mouse p-p38 (Proteintech Group Inc.), rabbit anti-mouse ERK1/2 (Proteintech Group Inc.), rabbit anti-mouse p-ERK1/2 (Proteintech Group Inc.), rabbit anti-mouse JNK (Proteintech Group Inc.), rabbit anti-mouse p-JNK (Proteintech Group Inc.), and mouse anti-*β*-actin (Abclonal Biotech Co.). Blots were washed with TBST solution for three times, every ten minutes, and then secondary antibodies were added for incubation and imaged using ECL detection reagents (Shanghai Jiapeng). The protein expression was quantified using the Image J (Shanghai Jiapeng) and Quantity One software. The signal intensity of the bands was normalized to that of the corresponding *β*-actin band.

### 2.6. Statistical Analysis

All data were expressed as mean ± SD. Statistical analyses were performed using GraphPad Instat software. The results were analyzed for statistical variance using an unpaired *t*-test or one-way ANOVA as appropriate. Results were considered statistically significant at *P* < 0.05.

## 3. Results

### 3.1. Hordenine Inhibited the Overgrowth of Pituitary Gland in Rats

**Figure 2:**
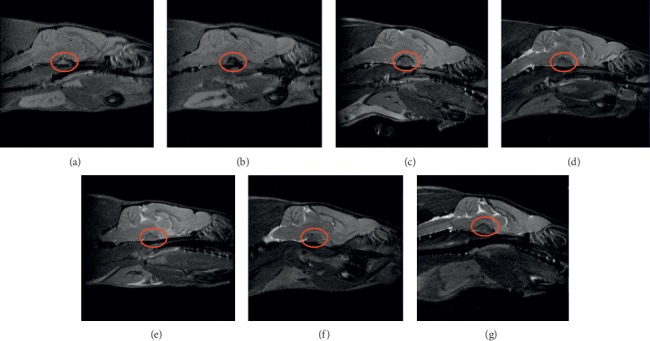
Pituitary gland imaging of rats by nuclear magnetic resonance: (a) control, (b) sham-operated control, (c) model control, (d) model + bromocriptine, (e) model + hordenine (152.8 mg/kg), (f) model + hordenine (76.4 mg/kg), and (g) model + hordenine (38.2 mg/kg).

**Figure 3:**
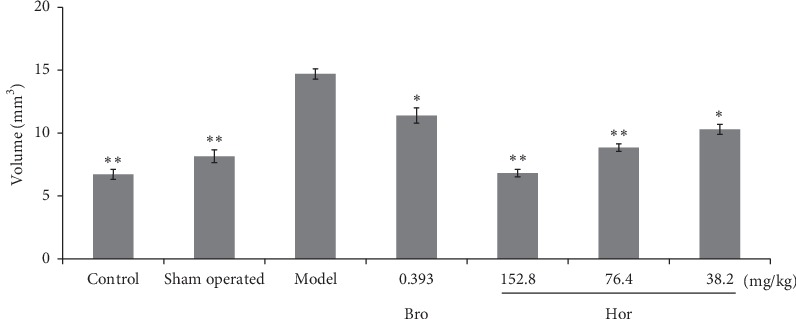
Pituitary gland volume of rats by nuclear magnetic resonance. Bro: bromocriptine; Hor: hordenine. Data were shown as mean ± SD (*n* = 10). ^*∗∗*^*P* < 0.01, ^*∗*^*P* < 0.05 compared with the model group.

Nuclear magnetic resonance imaging detection showed that, compared with the control and sham-operated control rats, estradiol increased the pituitary gland volume in rats significantly (*P* < 0.01). Hordenine succeeded in restoring the overgrowth of pituitary gland at 152.8 (*P* < 0.01), 76.4 (*P* < 0.01), and 38.2 mg/kg (*P* < 0.05) in this model. Bromocriptine at 0.393 mg/kg significantly inhibited the overgrowth of pituitary gland in rats (*P* < 0.05) (Figures 2 and 3).

### 3.2. Hordenine Inhibited the Serum Prolactin Level of Rats

**Figure 4:**
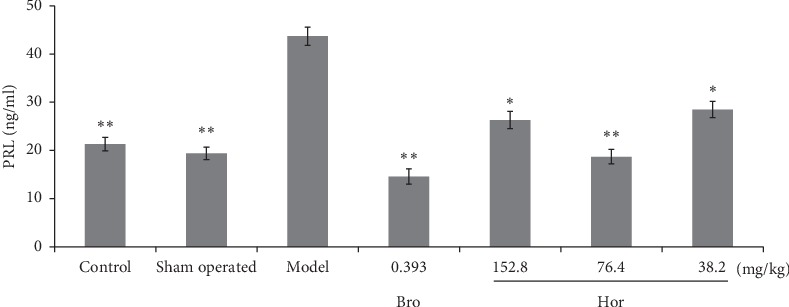
Serum prolactin level of rats by ELISA. Bro: bromocriptine; Hor: hordenine. Data were shown as mean ± SD (*n* = 8). ^*∗∗*^*P* < 0.01, ^*∗*^*P* < 0.05 compared with the model group.

Compared with the control and sham-operated control rats, estradiol significantly increased the serum prolactin level in rats (*P* < 0.01). Hordenine restored the increase of serum prolactin level at 152.8 (*P* < 0.05), 76.4 (*P* < 0.01), and 38.2 mg/kg (*P* < 0.05) in this model. Bromocriptine at 0.393 mg/kg significantly lowered the serum prolactin level in rats (*P* < 0.01) (Figure 4).

### 3.3. Hordenine Inhibited Prolactin Overproduction in Estradiol-Induced Rats through Regulating MAPK Signaling Pathway

**Figure 5:**
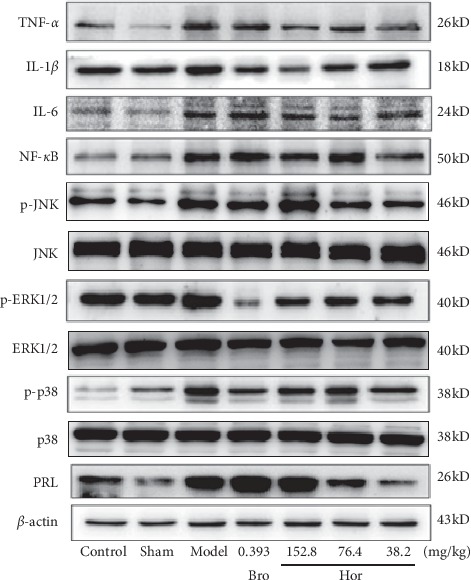
Western blot image of pituitary gland protein expressions in rats. Control: control group, Sham: sham-operated control group, Model: model control group, Bro: bromocriptine group, Hor: hordenine group.

**Figure 6:**
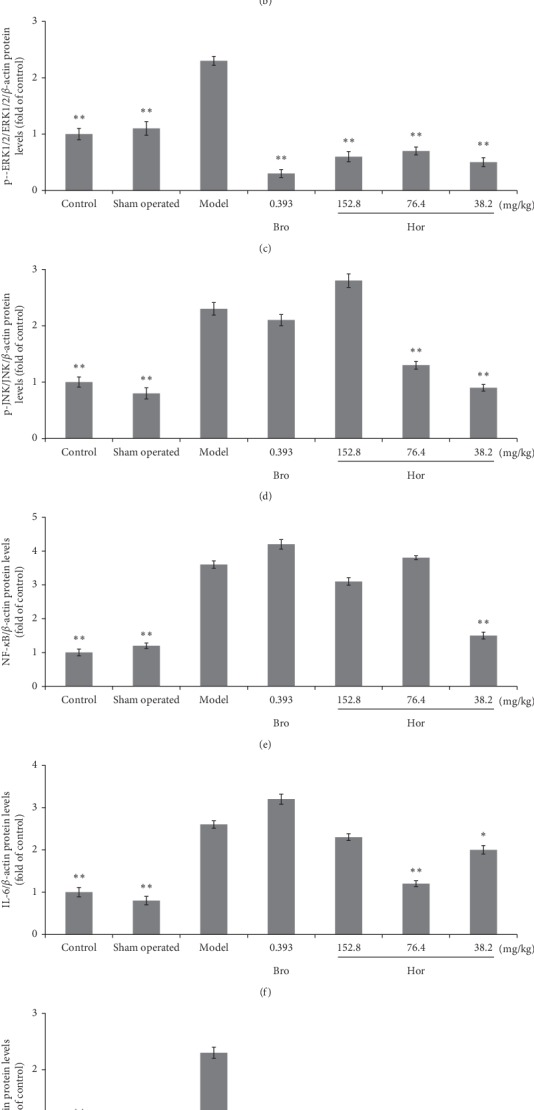
Protein levels of pituitary PRL (a), p-p38/p38 (b), p-ERK1/2/ERK1/2 (c), p-JNK/JNK (d), NF-*κ*B (e), IL-6 (f), IL-1*β* (g), and TNF-*α* (h) of rats were measured by western blot. Data were shown as mean ± SD (*n* = 4). Compared with the model group, ^*∗∗*^*P* < 0.01, ^*∗*^*P* < 0.05.

Compared with the control and sham-operated control rats, estradiol significantly increased pituitary gland protein level of PRL (*P* < 0.01), p-p38 (*P* < 0.01), p-ERK1/2 (*P* < 0.01), p-JNK (*P* < 0.01), NF-*κ*B (*P* < 0.01), TNF-*α* (*P* < 0.01), IL-1*β* (*P* < 0.01), and IL-6 (*P* < 0.01) in rats. Hordenine at 38.2 mg/kg significantly downregulated the protein level of the pituitary gland PRL (*P* < 0.01), p-p38 (*P* < 0.01), p-ERK1/2 (*P* < 0.01), p-JNK (*P* < 0.01), NF-*κ*B (*P* < 0.01), TNF-*α* (*P* < 0.01), IL-1*β* (*P* < 0.01), and IL-6 (*P* < 0.01), at 76.4 mg/kg lowered pituitary gland protein level of PRL (*P* < 0.01), p-ERK1/2 (*P* < 0.01), p-JNK (*P* < 0.01), TNF-*α* (*P* < 0.01), IL-1*β* (*P* < 0.01), and IL-6 (*P* < 0.01), and at 152.8 mg/kg only lowered p-ERK1/2 (*P* < 0.01), TNF-*α* (*P* < 0.01), and IL-1*β* (*P* < 0.01) in estradiol-induced rats. And, bromocriptine also succeeded in restoring estradiol-induced overexpression of pituitary gland protein level of p-p38 (*P* < 0.05), p-ERK1/2 (*P* < 0.01), and IL-1*β* (*P* < 0.01) at 0.393 mg/kg in this model (Figures 5 and 6).

## 4. Discussion

As one of the complementary and alternative medicine options, traditional Chinese herbal drug is currently commonly used and popular in China. Fructus Hordei Germinatus has been used to cure hyperprolactinemia for thousands of years. Hordenine, an alkaloid and a major constituent of Fructus Hordei Germinatus, has been previously demonstrated to possess antihyperprolactinemia effects [32]. It attracted our attention for further development. Thus, this study investigated the effect and mechanism of hordenine on prolactinomas induced by estradiol in rats. We used F344 rats to prepare the model and demonstrated that hordenine restored the overgrowth of pituitary gland and reduced the PRL accumulation in the serum and pituitary gland of rats. The results confirmed that hordenine may improve the abnormalities of prolactinomas.

We then studied the possible anti-prolactinomas mechanism of hordenine in rats. MAPK is an important transmitter of signal from cell surface to nucleus, which is followed by phosphorylation of many kinds of cytosolic proteins associated with cell proliferation, cell invasion, cell differentiation, cell migration, and cell apoptosis [33]. MAPK is composed of p38 MAPK, c-jun-N-terminal kinase (JNK1/2), and extracellular signal-regulated kinase (ERK1/2) [12]. The p38 MAPK is stimulated and activated by various kinds of cellular stresses, such as lipopolysaccharides (LPS), ultraviolet light, inflammatory cytokines, and growth factors and then makes this signaling pathway a potential therapeutic target for inflammatory diseases [34]. The p38 MAPK have tumorigenic functions in certain contexts [35]. We used western blotting to analyze the effects of hordenine on MAPK protein expressions in estradiol-induced prolactinomas rats. The western blotting results showed that estradiol significantly increased pituitary gland protein levels of PRL, p-p38, p-ERK1/2, p-JNK, NF-*κ*B, TNF-*α*, IL-1*β*, and IL-6 in rats, which were reversed by hordenine treatment. Our results suggested that the the MAPK pathway and cytokines/ILs were significantly elevated in the progress of prolactinoma model, and hordenine inhibited the production of PRL by regulating MAPKs signaling. However, the moleclular mechanism of the MAPK pathway and cytokines/ILs in the prolactinomas model is not very clear and is being studied by our group.

At present, the clinical first-line treatment drugs for prolactinomas are bromocriptine and cabergoline, which are dopamine D2 receptor agonists. However, 25% of patients do not respond to this therapy, and there are obvious side effects such as nausea. Thus, it is necessary to study new targets and drugs for the treatment of prolactinomas. The present study showed that 30 days of treatment of hordenine inhibited the overgrowth of pituitary glands, reduced the prolactin expression in the serum and pituitary gland, and decreased the expression of p-p38, p-ERK1/2, p-JNK, NF-*κ*B, TNF-*α*, IL-1*β*, and IL-6 of pituitary glands in rats. Based on the findings of this study, it suggests that there is a regulatory effect of hordenine on MAPK signaling. We supposed that the antiprolactinoma-like effects of hordenine are mediated by inhibiting the MAPK signaling activation in estradiol-induced rats.

## 5. Conclusion

In conclusion, the present study showed that hordenine (76.4 and 152.8 mg/kg) inhibited the overgrowth and PRL expression of the pituitary gland in estradiol-induced rats via regulation of the MAPK pathway, lowering the p-p38, p-ERK1/2, and p-JNK protein expression and the production of inflammatory cytokines TNF-*α*, IL-1*β*, and IL-6, which provided the new therapeutic targets and drugs for treating prolactinomas.

## Data Availability

The data used to support the findings of this study are included within the article.
